# Association of Childhood Physical and Sexual Abuse with Intimate Partner Violence, Poor General Health and Depressive Symptoms among Pregnant Women

**DOI:** 10.1371/journal.pone.0116609

**Published:** 2015-01-30

**Authors:** Yasmin V. Barrios, Bizu Gelaye, Qiuyue Zhong, Christina Nicolaidis, Marta B. Rondon, Pedro J. Garcia, Pedro A. Mascaro Sanchez, Sixto E. Sanchez, Michelle A. Williams

**Affiliations:** 1 Department of Epidemiology, Harvard School of Public Health, Boston, MA, United States of America; 2 School of Social Work, Portland State University, Portland, OR, United States of America; 3 Department of Medicine, Section on Psychiatry and Mental Health, Cayetano Heredia Peruvian University, Lima, Peru; 4 Instituto Nacional Materno Perinatal, Lima, Peru; 5 Universidad Peruana de Ciencias Aplicadas, Lima, Peru; 6 Asociación Civil PROESA, Lima, Peru; Univ of Toledo, UNITED STATES

## Abstract

**Objective:**

We examined associations of childhood physical and sexual abuse with risk of intimate partner violence (IPV). We also evaluated the extent to which childhood abuse was associated with self-reported general health status and symptoms of antepartum depression in a cohort of pregnant Peruvian women.

**Methods:**

In-person interviews were conducted to collect information regarding history of childhood abuse and IPV from 1,521 women during early pregnancy. Antepartum depressive symptomatology was evaluated using the Patient Health Questionnaire-9. Multivariable logistic regression procedures were used to estimate adjusted odds ratios (aOR) and 95% confidence intervals (95%CI).

**Results:**

Any childhood abuse was associated with 2.2-fold increased odds of lifetime IPV (95%CI: 1.72–2.83). Compared with women who reported no childhood abuse, those who reported both, childhood physical and sexual abuse had a 7.14-fold lifetime risk of physical and sexual IPV (95%CI: 4.15–12.26). The odds of experiencing physical and sexual abuse by an intimate partner in the past year was 3.33-fold higher among women with a history of childhood physical and sexual abuse as compared to women who were not abused as children (95%CI 1.60–6.89). Childhood abuse was associated with higher odds of self-reported poor health status during early pregnancy (aOR = 1.32, 95%CI: 1.04–1.68) and with symptoms of antepartum depression (aOR = 2.07, 95%CI: 1.58–2.71).

**Conclusion:**

These data indicate that childhood sexual and physical abuse is associated with IPV, poor general health and depressive symptoms in early pregnancy. The high prevalence of childhood trauma and its enduring effects of on women’s health warrant concerted global health efforts in preventing violence.

## Introduction

Childhood abuse has been shown to be associated with serious adverse health consequences across the life course [1–4]. Notably, childhood trauma and adverse experiences have been linked with increased risks of developmental difficulties, sensation seeking behaviors, as well as somatic and mental health outcomes including increased risks of asthma [5], early age at menarche [6,7] chronic systemic inflammation [8], substance abuse [9], mood and anxiety disorders [10], suicidal behaviors [2] and premature mortality [11]. Some investigative teams have reported that women with a history of childhood abuse, particularly childhood sexual abuse, may be at exceptionally high risk of revictimization in adulthood [12,13]. For example, Russell et al [12] reported that childhood incest victims were almost twice as likely to experience rape or attempted rape after age 14 years, as compared with a group of women with no history of incest. Similar associations of childhood abuse with increased risks of both physical and sexual abuse have been documented in population-based surveys, clinical samples and cohorts of college women [14,15]. Additionally, a history of childhood abuse has been linked to an increased risk of exposure to intimate partner violence (IPV) in adulthood by most [16–20], though not all [21] previous investigators.

Despite inconsistent findings across studies, available evidence suggests that women with a history of childhood abuse have an increased risk of IPV in adulthood. Many of the earlier studies, however, have been conducted in North American and European populations. Hence, relatively little is known about the epidemiology and association of childhood abuse and IPV among women in low- and middle-income countries such as Peru. In the recent World Health Organization [22] multi-country study on domestic violence, the lifetime prevalence of any physical or sexual partner violence varied from 15% to 71% [23–25]. Moreover, studies have also shown that victims of IPV are more likely to experience depression [26–28], anxiety [29], suicide ideation [30–33], and post-traumatic stress disorder [34–36] than those without such history. However, many studies have not quantified risk by type of abuse experienced at the hands of an intimate partner in relation to type of childhood abuse. In a large sample of low-income pregnant Peruvian women receiving routine prenatal care, we examined the associations of women’s childhood experiences of physical and sexual abuse with risk of physical and sexual abuse by an intimate partner. We also evaluated the extent to which, if at all, childhood physical and sexual abuse was associated with women’s self-reported general health status and symptoms of antepartum depression.

## Methods

### The PrOMIS Study

The population for the present study was drawn from participants of the ongoing Pregnancy Outcomes, Maternal and Infant Study (PrOMIS) Cohort, designed to examine maternal social and behavioral risk factors of preterm birth and other adverse pregnancy outcomes among Peruvian women. The study population consists of women attending prenatal care clinics at the Instituto Nacional Materno Perinatal (INMP) in Lima, Peru. The INMP is the primary reference establishment for maternal and perinatal care operated by the Ministry of Health of the Peruvian government. Recruitment began in February 2012. Women eligible for inclusion were those who initiated prenatal care prior to 16 weeks gestation since, on average, less than 10% of women initiate prenatal care after 16 weeks of gestation at INMP. Women were ineligible if they were younger than 18 years of age, did not speak and read Spanish, or had completed more than 16 weeks gestation.The eligibility criteria threshold of initiating prenatal care prior to the completion of 16 weeks gestation was set so as to mitigate concerns about reverse causality and recall bias while enrolling a study population that is sufficiently generalizable to the source population of women seeking care at the study site. Before setting this threshold, we determined that over 90% of women delivering at INMP initiate prenatal care prior to 16 weeks gestation.

Enrolled participants were invited to take part in an interview where trained research personnel used a structured questionnaire to elicit information regarding maternal socio-demographic, lifestyle characteristics, medical and reproductive histories, and early life experiences of abuse and with symptoms of mood and anxiety disorders. All participants provided written informed consent. The institutional review boards of the INMP, Lima, Peru and the Harvard School of Public Health Office of Human Research Administration, Boston, MA approved all procedures used in this study.

### Analytical Population

The study population for this report is derived from information collected from those participants who enrolled in the PrOMIS Cohort Study between February 2012 and March 2013. During this period 1,810 eligible women were approached, and 1,556 (86%) agreed to participate. Thirty-five participants were excluded from the present analysis because of missing information concerning experience with abuse in childhood and/or missing information for IPV in adulthood. Women excluded from this analysis did not differ in regards to sociodemographic and lifestyle characteristics as compared with those included. A total of 1,521 women remained for analysis.

### Childhood Abuse Assessment

We used the Childhood Physical and Sexual Abuse Questionnaire to elicit information concerning participants’ experiences with physical and sexual abuse in childhood [37]. The instrument consists of eight questions concerning abuse taken from the Centers for Disease Control and Prevention (CDC) Adverse Childhood Experiences Study. Participants were rated as having experienced childhood abuse if, before the age of 18 years, they reported that an older person touched them, they were made to touch someone else in a sexual way, or intercourse was attempted or completed (sexual abuse); or that they were hit, kicked, or beaten often and/or their life was seriously threatened (physical abuse). Participants who responded ‘no’ to all questions regarding sexual and physical abuse were categorized as ‘no abuse’. Those responding ‘yes’ to only physical abuse questions were categorized as ‘physical abuse only’ and those responding ‘yes’ to only sexual abuse questions were categorized as ‘sexual abuse only’. Those responding ‘yes’ to any physical abuse questions and ‘yes’ to any sexual abuse questions were categorized as having experienced ‘both physical and sexual abuse’. Participants who responded ‘yes’ to any questions of physical abuse or ‘yes’ to any questions of sexual abuse or yes to both abuse types were categorized as having experienced ‘any abuse’.

### IPV Assessment

Questions on IPV were adapted from the protocol of Demographic Health Survey Questionnaires and Modules: Domestic Violence Module [38] and the WHO Multi-Country Study on Violence Against Women [23]. Women were assessed for a range of physical and/or sexual coercive acts used against them by a current or former husband or intimate partner without their consent. Women were classified as having experienced moderately severe physical violence if they endorsed any of the following acts: being slapped, having her arms twisted or something thrown at her, being pushed or shoved. Participants were classified as having experienced severe physical violence if they reported experiencing any of the following acts: being hit, kicked, dragged or beaten up, being choked or burnt on purpose, or being threatened or hurt with a weapon (such as, gun, knife, or other object). Participants were classified as having experienced sexual violence if they endorsed any of the following: being physically forced to have sexual intercourse, having had unwanted sexual intercourse because of fear of what the partner might do, and being forced to perform other sexual acts that the respondent found degrading or humiliating. In this study, women were categorized as having experienced one or more acts of physical or sexual violence, physical violence only, sexual violence only, or both physical and sexual violence at any time from a current or former male partner. All study personnel were trained on interviewing skills, contents of the questionnaire, and ethical conduct of violence research (including issues of safety and confidentiality). Interviewers were trained to refer participants found to be in physically dangerous situations and/or in immediate need for counseling to psychologists at local women’s organizations, hospital psychiatrists, and battered women’s shelters.

### Depressive Symptoms

Depressive symptomatology during pregnancy was evaluated using the Patient Health Questionnaire-9 (PHQ-9) [39]. The PHQ-9 has been demonstrated to be a reliable tool for assessing depressive disorders among a diverse group of obstetrics-gynecology patients [40,41] and in Spanish-speaking women [42]. The 9-item instrument asks respondents to rate the relevancy of each statement comprising emotional, cognitive, and functional somatic symptoms over the past two weeks on a four-point scale a) never; b) several days; c) more than half the days; or d) nearly everyday. The PHQ-9 total score is the sum of scores for the nine items for each participant, and ranged from 0–27. Participants were assigned to one of five depressive symptom categories based on total PHQ-9 score, (a) no depressive symptoms (0–4), (b) mild (5–9), (b) moderate (10–14), (c) moderately severe (15–19) and (d) severe (20–27) depressive symptoms. For the purpose of this study, we assigned participants to one of two categories of depressive symptoms based on total PHQ-9 score, (a) no depressive symptoms (0–9) and (b) moderate to severe depressive symptoms (10–27) [39,43,44]. A meta-analysis of 14 studies support the use of a PHQ-9 score of ≥10 to classify subjects with major depressive disorder [45]. Briefly, the authors reported that a cut-off of ≥10 had a sensitivity and specificity of 0.80 and 0.92, respectively [45].

### Other Covariates

Participants’ age was categorized as follows: 18–20, 20–29, 30–34, and ≥35 years. Other sociodemographic variables were categorized as follows: maternal ethnicity (Hispanic vs. others); educational attainment (≤6, 7–12, and >12 completed years of schooling); marital status (married and living with partner vs. others); employment status (employed vs. not employed); access to basic foods (very hard/hard, somewhat hard, not very hard); parity (nulliparous vs. multiparous); planned pregnancy (yes vs. no); self-reported health in the last year (good vs. poor) and gestational age at interview.

### Statistical Analyses

Frequency distributions of maternal sociodemographic and reproductive characteristics were examined. Chi-square tests for categorical variables and Student’s *t* tests for continuous variables were conducted to determine whether there were statistically significant differences in the association between socio demographic and reproductive characteristics and history of any childhood abuse. Multivariate adjusted logistic regression procedures were used to calculate maximum likelihood estimates of odds ratios (ORs) and 95% confidence intervals (CIs) of history of lifetime IPV (any lifetime physical or sexual violence, physical violence only, sexual violence only, and both physical and sexual violence) in relation to history of childhood abuse. We included covariates of *a priori* interest (i.e., maternal age, education, employment status, parity and difficulty paying for the very basics) in the final multivariate adjusted logistic regression models. Multinomial logistic regression procedures were used to estimate odds of lifetime IPV in relation to types of history of childhood abuse (e.g., physical abuse only, sexual abuse only, and both physical and sexual abuse). These analyses were important for identifying heterogeneity in risk of IPV in relation to prior history of abuse. For these analyses, women who had no history of childhood abuse (either physical or sexual) and no history of IPV constituted the reference group. All statistical analyses were performed using SAS 9.3 (SAS Institute, Cary, NC, USA). All reported *P*-values are 2-tailed with statistical significance set at 0.05.

## Results

**Figure 1:**
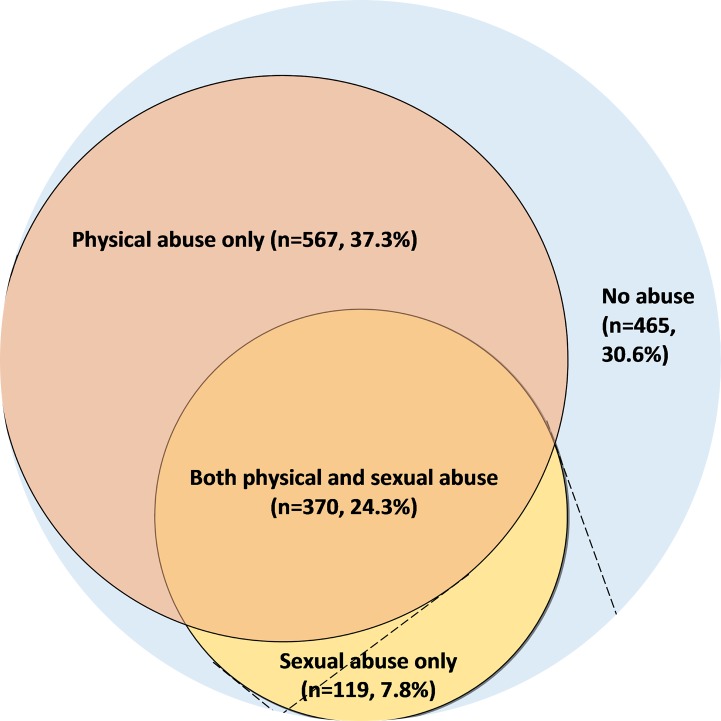
Prevalence of Childhood Physical and Sexual Abuse by Type and Perpetrators among Pregnant Women in Lima, Peru (N = 1,521).

In this cohort, the vast majority of participants reported exposure to physical or sexual abuse as a child. As shown in **Fig. 1**, 61.1% of exposed women reported experience of physical abuse; and 32.2% reported experience with sexual abuse. Overall, 37.3% of the cohort reported childhood physical abuse only, 24.3% of the cohort reported experience with both physical and sexual abuse; and 7.8% experienced sexual abuse only in childhood. Of the 489 women who experienced sexual abuse in childhood, 24% were victims of rape. Additionally, of the 489 women who were sexually abused in childhood, 93.3% reported that an adult sexually abused them.

Sociodemographic and reproductive characteristics of the study population are summarized in **Table 1**. Approximately 70.0% of study participants reported a history of physical or sexual abuse in childhood (<18 years of age). Individuals who were exposed to physical or sexual abuse as a child were older, more likely to have difficulty paying for basic needs, and to report poor health status as compared with those who were not abused as a child. Participants who were abused as children were less likely to be nulliparous as compared to those not abused as a child. The two study groups were similar with regards to educational attainment, gestational age at interview, race/ethnicity and employment status. Of note, individuals abused in childhood were more likely to have a positive lifetime history of physical or sexual abuse by an intimate partner in adulthood (44.9% vs. 25.8%, p-value <0.01).

**Table 1:** Sociodemographics and reproductive characteristics of the study population.

**Characteristic**	**Participants (N = 1521)**		**Childhood Physical or Sexual Abuse**				
			**Yes**		**No**		
			**(N = 1056)**		**(N = 465)**		***P* value**
	**n**	**%**	**n**	**%**	**n**	**%**	
Age (years)*	28.0 ± 6.2		28.3 ± 6.2		27.3 ± 6.2		<0.01
Age (years)							
18–20	88	5.8	59	5.6	29	6.2	0.03
20–29	869	57.1	579	54.8	290	62.4	
30–34	300	19.7	222	21.0	78	16.8	
≥35	264	17.4	196	18.6	68	14.6	
Education (years)							
≤6	68	4.5	47	4.5	21	4.5	0.93
7–12	852	56.0	588	55.7	264	56.8	
>12	596	39.2	417	39.5	179	38.5	
Mestizo	1143	75.2	788	74.6	355	76.3	0.41
Married/living with a partner	1237	81.3	853	80.8	384	82.6	0.48
Employed	660	43.4	461	43.7	199	42.8	0.74
Access to basic foods							
Very hard/hard	281	18.5	216	20.5	65	14.0	<0.01
Somewhat hard	492	32.4	357	33.8	135	29.0	
Not very hard	747	49.1	482	45.6	265	57.0	
Nulliparous	765	50.3	503	47.6	262	56.3	<0.01
Planned pregnancy	640	42.1	435	41.2	205	44.1	0.53
Gestational age at interview*	9.8 ± 3.4		9.8 ± 3.3		9.6 ± 3.4		0.15
Self-reported health status in last year							
Good	1043	68.6	690	65.3	353	75.9	
Poor	453	29.8	349	33.1	104	22.4	
Self-reported health status during pregnancy							
Good	483	31.8	313	29.6	170	36.6	0.01
Poor	990	65.1	709	67.1	281	60.4	
Depression**	442	29.1	352	33.3	90	19.4	<0.01
Any lifetime sexual or physical abuse by intimate partner							
No	927	61.0	582	55.1	345	74.2	<0.01
Yes	594	39.1	474	44.9	120	25.8	

Due to missing data, percentages may not add up to 100%.

*mean ± SD (standard deviation)

** Depression is defined as a score ≥10 on PHQ-9 scale

**Table 2**, shows adjusted odds ratio (aOR) and 95% confidence interval (95% CI) of experiencing lifetime IPV in relation to childhood abuse history. Women with a history of experiencing any childhood abuse had 2.2-fold increased odds of suffering from any lifetime IPV (aOR = 2.20; 95%CI: 1.72–2.83). Compared to women who reported no history of childhood abuse those who experienced any childhood abuse also had increased odds of experiencing lifetime physical IPV only (aOR = 1.94; 95%CI: 1.45–2.58), lifetime sexual IPV only (aOR = 2.27; 95%CI: 1.30–3.95) and lifetime physical and sexual IPV (aOR = 3.29; 95%CI: 2.01–5.38). The association of experiencing IPV during lifetime was particularly strong among women with a history of both physical and sexual abuse during childhood (aOR = 7.14; 95%CI: 4.15–12.26) lifetime IPV. Associations of childhood abuse with IPV during the past year were similar in direction as lifetime IPV, but lower in magnitude (**Table 3**). Compared to women who had no childhood abuse history, those women who suffered from both physical and sexual childhood abuse had the highest odds of experiencing any IPV (aOR = 3.00; 95%CI: 2.07–4.35), physical IPV only (aOR = 2.73; 95%CI: 1.77–4.20), sexual IPV only (aOR = 4.24; 95%CI: 1.77–10.17) and both physical and sexual IPV (aOR = 3.33; 95%CI: 1.60–6.89) in the past year.

**Table 2:** Risk of intimate partner violence *during lifetime* according to reports of childhood abuse experience* (N = 1, 521).

**Childhood Abuse**	**No IPV N = 927**	**Any Lifetime IPV N = 594**		**Lifetime IPV Physical Abuse N = 363**		**Lifetime IPV Sexual Abuse N = 84**		**Lifetime IPV Physical and Sexual Abuse N = 147**	
	**n (%)**	**n (%)**	**aOR (95% CI)**	**n (%)**	**aOR (95% CI)**	**n (%)**	**aOR (95% CI)**	**n (%)**	**aOR (95% CI)**
No Abuse	345 (37.22)	120 (20.20)	Reference	82 (22.59)	Reference	17 (20.24)	Reference	21 (14.29)	Reference
Any Abuse	582 (62.78)	474 (79.80)	**2.20 (1.72–2.83)**	281 (77.41)	**1.94 (1.45–2.58)**	67 (79.76)	**2.27 (1.30–3.95)**	126 (85.71)	**3.29 (2.01–5.38)**
Types of Abuse									
No Abuse	345 (37.22)	120 (20.20)	Reference	82 (22.59)	Reference	17 (20.24)	Reference	21 (14.29)	Reference
Physical Abuse Only	361 (38.94)	206 (34.68)	**1.57 (1.19–2.07)**	145 (39.94)	**1.63 (1.19–2.24)**	22 (26.19)	1.19 (0.62–2.30)	39 (26.53)	1.64 (0.93–2.89)
Sexual Abuse Only	68 (7.34)	51 (8.59)	**2.14 (1.39–3.29)**	30 (8.26)	**1.88 (1.13–3.13)**	7 (8.33)	2.09 (0.83–5.31)	14 (9.52)	**3.36 (1.59–7.10)**
Physical & Sexual Abuse	153 (16.50)	217 (36.53)	**3.73 (2.75–5.04)**	106 (29.20)	**2.68 (1.88–3.83)**	38 (45.24)	**5.00 (2.71–9.22)**	73 (49.66)	**7.14 (4.15–12.26)**

CI = confidence interval; aOR = adjusted odds ratio; Bold = statistically significant values.

*Odds ratio adjusted for maternal age (years), education (years), employment status (yes vs. no), parity (nulliparous vs. multiparous) and difficulty paying for the very basics (very hard or hard, somewhat hard, not very hard)

**Table 3:** Risk of intimate partner violence during *the past year* according to reports of childhood abuse experiences* (N = 1, 226).

**Childhood Abuse**	**No IPV (N = 937)**	**Any Past Year IPV (N = 289)**		**Past Year IPV Physical Abuse (N = 196)**		**Past Year IPV Sexual Abuse (N = 38)**		**Past Year IPV Physical and Sexual Abuse (N = 55)**	
	**n (%)**	**n (%)**	**aOR (95% CI)**	**n (%)**	**aOR (95% CI)**	**n (%)**	**aOR (95% CI)**	**n (%)**	**aOR (95% CI)**
No Abuse	345(36.82)	68(23.53)	Reference	47(23.98)	Reference	8(21.05)	Reference	13(23.64)	Reference
Any Abuse	592(63.18)	221(76.47)	**1.79 (1.31, 2.44)**	149(76.02)	**1.76 (1.23, 2.53)**	30(78.95)	2.05 (0.92, 4.55)	42(76.36)	1.73 (0.90, 3.32)
Types of abuse									
No Abuse	345(36.82)	68(23.53)	Reference	47(23.98)	Reference	8(21.05)	Reference	13(23.64)	Reference
Physical Abuse Only	364(38.85)	89(30.80)	1.20 (0.84, 1.71)	65(33.16)	1.27 (0.84, 1.91)	10(26.32)	1.13 (0.44, 2.91)	14(25.45)	0.95 (0.44, 2.08)
Sexual Abuse Only	68(7.26)	28(9.69)	**2.05 (1.21, 3.48)**	20(10.20)	**2.14 (1.16, 3.92)**	3(7.89)	1.82 (0.46, 7.15)	5(9.09)	2.08 (0.70, 6.18)
Physical & Sexual Abuse	160(17.08)	104(35.99)	**3.00 (2.07, 4.35)**	64(32.65)	**2.73 (1.77, 4.20)**	17(44.74)	**4.24 (1.77, 10.17)**	23(41.82)	**3.33** **(1.60, 6.89)**

CI = confidence interval; aOR = adjusted odds ratio; Bold = statistically significant values.

* Odds ratio adjusted for maternal age (years), education (years), employment status (yes vs. no), parity (nulliparous vs. multiparous) and difficulty paying for the very basics (very hard or hard, somewhat hard, not very hard)

**Table 4**, shows the association of childhood abuse with self-reported health status in the past year and during the current pregnancy. Compared to women with no childhood abuse history, those women who experienced any childhood abuse had increased odds of reporting their health as poor in the past year (aOR = 1.63; 95%CI: 1.26–2.11) and during the current pregnancy (aOR = 1.32; 95%CI: 1.04–1.68). We found elevated odds of poor self-reported health in the past year among abused women, specifically for women with a history of both physical and sexual childhood abuse (aOR = 2.12; 95%CI: 1.56–2.89). Of note, the association remained virtually unchanged when we included women’s experience with IPV in the current pregnancy into the model (aOR = 2.02; 95% CI: 1.42–2.87). A similar association, but lower in magnitude, was found for self-reported poor health status during the current pregnancy.

**Table 4:** Risk of poor general health according to reports of childhood abuse experience*.

**Childhood Abuse**	**Health in Last Year**					**Health during This Pregnancy**				
	Excellent/Good** (N = 1043)		Poor** (N = 453)			Excellent/Good** (N = 483)		Poor** (N = 990)		
	**n**	**(%)**	**n**	**(%)**	**aOR (95% CI)**	**n**	**(%)**	**n**	**(%)**	**aOR (95% CI)**
No Abuse	353	(33.84)	104	(22.96)	Reference	170	(35.20)	281	(28.38)	Reference
Any Abuse	690	(66.16)	349	(77.04)	**1.63 (1.26–2.11)**	313	(64.80)	709	(71.62)	**1.32 (1.04–1.68)**
Types of Abuse										
No Abuse	353	(33.84)	104	(22.96)	Reference	170	(35.20)	281	(28.38)	Reference
Physical Abuse Only	396	(37.97)	164	(36.2)	**1.36 (1.02–1.81)**	193	(39.96)	362	(36.57)	1.12 (0.86–1.45)
Sexual Abuse Only	77	(7.38)	40	(8.83)	**1.69 (1.08–2.65)**	39	(8.07)	75	(7.58)	1.17 (0.75–1.82)
Physical & Sexual Abuse	217	(20.81)	145	(32.01)	**2.12 (1.56–2.89)**	81	(16.77)	272	(27.47)	**1.88 (1.36–2.59)**

CI = confidence interval; aOR = adjusted odds ratio; Bold = statistically significant values.

* Odds ratio adjusted for maternal age (years), education (years), employment status (yes vs. no), parity (nulliparous vs. multiparous) and difficulty paying for the very basics (very hard or hard, somewhat hard, not very hard)

**Excellent/Good health is defined as excellent or very good or good health. Poor health is defined as fair or poor health.

We also evaluated the prevalence of antepartum depression, measured using the PHQ-9 instrument, according to childhood abuse. As shown in **Table 5**, compared to women who had no childhood experience with physical or sexual abuse, those who experienced any childhood abuse had 2.1-fold increased odds of experiencing antepartum depression (aOR = 2.07; 95%CI: 1.58–2.71). We observed elevated odds of antepartum depression among women with a history of both physical and sexual childhood abuse (aOR = 2.47; 95%CI: 1.79–3.40); and this association remained even when exposure to IPV during the index pregnancy was accounted for in multivariable models (aOR = 2.14; 95%CI: 1.47–3.11). Of note, the odds of antepartum depression were not elevated in women with a history of only sexual childhood abuse.

**Table 5:** Risk of depressive symptoms according to reports of childhood abuse experiences* (N = 1,503).

**Childhood Abuse**	**No Depression (N = 1061)**		Depression** (N = 442)		
	**n**	**(%)**	**n**	**%**	**aOR (95% CI)**
No Abuse	371	(34.97)	90	(20.36)	Reference
Any Abuse	690	(65.03)	352	(79.64)	**2.07 (1.58–2.71)**
Types of Abuse					
No Abuse	371	(34.97)	90	(20.36)	Reference
Physical Abuse Only	373	(35.16)	186	(42.08)	**2.05 (1.53–2.75)**
Sexual Abuse Only	91	(8.58)	27	(6.11)	1.20 (0.73–1.96)
Physical & Sexual Abuse	226	(21.30)	139	(31.45)	**2.47 (1.79–3.40)**

CI = confidence interval; aOR = adjusted odds ratio; Bold = statistically significant values.

* Odds ratio adjusted for maternal age (years), education (years), employment status (yes vs. no), parity (nulliparous vs. multiparous) and difficulty paying for the very basics (very hard or hard, somewhat hard, not very hard)

**presence of antepartum depression is defined as a score ≥10 on PHQ-9 scale

**Figure 2:**
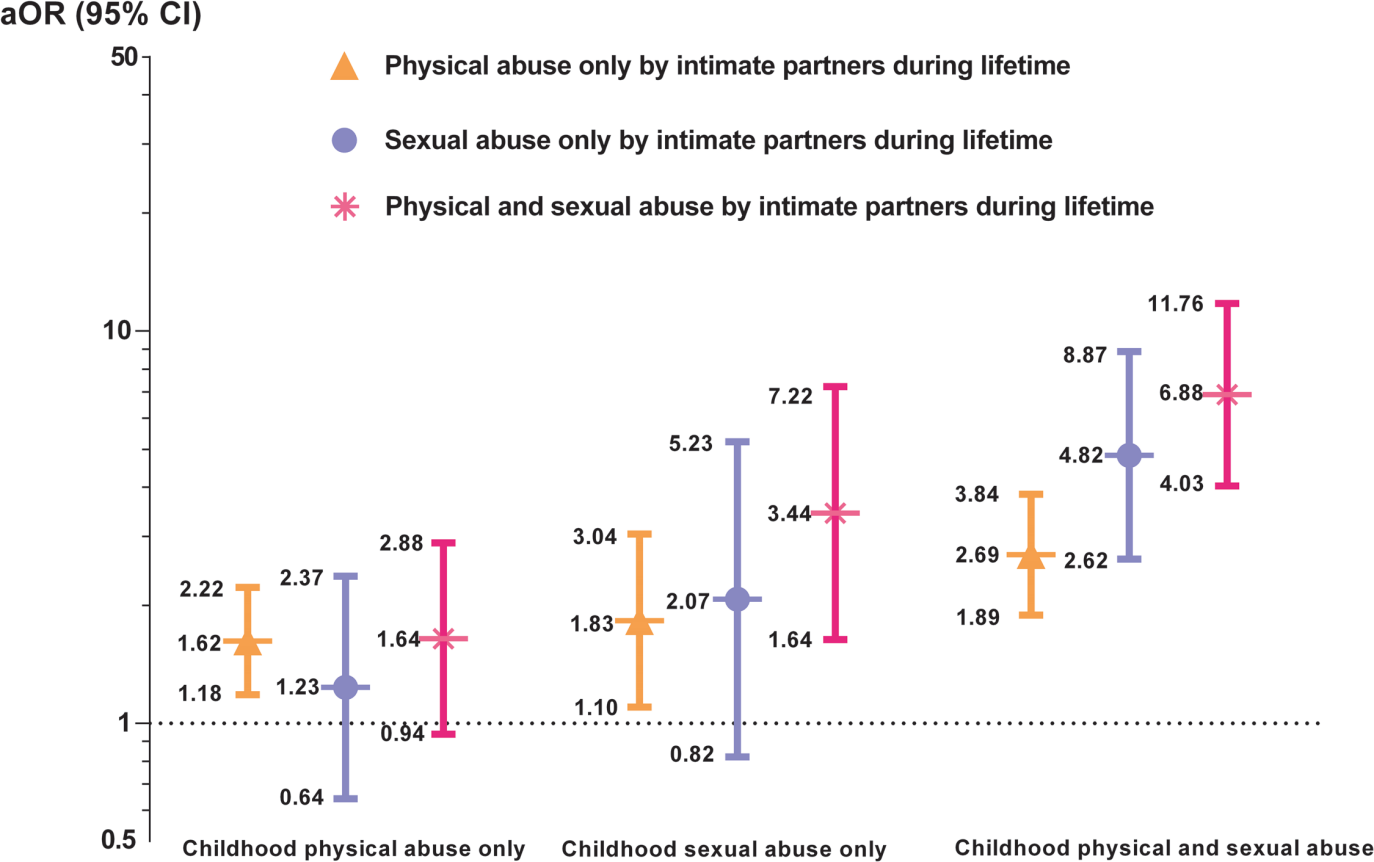
Adjusted Odds Ratios (aOR) and 95% Confidence Interval (CI) of Being Abused by Intimate Partners during Lifetime in Relation to Childhood Abuse among Women in Lima, Peru (n = 1,521).*

Finally, we wanted to evaluate the phenomenon of re-victimization, and therefore repeated multinomial logistic regression to identify whether women who reported childhood physical only, sexual abuse only or both physical and sexual abuse had distinctly different odds of experiencing subsequent intimate partner violence in adulthood. In **Fig. 2** we can see that the odds of revictimization by an intimate partner increased for all women who experienced any childhood abuse. The association was particularly strong for those who experienced both physical and sexual abuse. For example, compared to women never abused, those women who experience childhood sexual abuse only had a 3.4-fold increased odds of suffering physical and sexual abuse IPV during lifetime (OR = 3.44; 95%CI: 1.64–7.22). Notably, those who experienced both types of childhood abuse, physical and sexual, had a 6.9-fold increased odds of subsequently also experiencing both types of adulthood IPV (aOR = 6.88; 95%CI: 4.03–11.76). These results indicate that women with a history of childhood abuse, particularly those physically and sexually abused, are at increased risk for revictimization by an intimate partner during their lifetime.

## Discussion

Our study extends the literature by adding evidence of increased odds of adult physical and sexual abuse among pregnant Peruvian women with a history of childhood physical and sexual abuse. Strengths of our study include having a large sample size, the relatively high participation rate (86%), and the use of structured questionnaires including a validated instrument for assessing maternal antepartum depression. Furthermore, multivariable regression analyses are conducted to evaluate the type/types of childhood abuse that are associated with IPV, health status and antepartum depression. Because exposure to physical and sexual abuse is highly interrelated, we conducted statistical analyses to simultaneously consider the impact of independent and joint experiences on each outcome.

Several limitations, however, should be considered when interpreting the results from our study. First, experience of childhood abuse, IPV, and maternal antepartum depressive symptoms were assessed based on self-report in this cross-sectional study. Therefore, these measures may be subjected to non-systematic errors in recall, as well as systematic non-disclosure leading to misclassification. Investigators have noted that individuals are likely to minimize experiences of past violence rather than suggest that they had experienced violence in their lifetime [46]. Errors in recall may have led to an underestimation of reported associations. Indeed, investigators who have conducted longitudinal studies of adults whose childhood abuse was documented have reported that participants’ retrospective reports of childhood abuses are likely to underestimate actual experiences [47,48]. In addition, since the exact age of onset for childhood abuse was not collected in our study, it is possible that younger study participants whose onset was closer to the interview period might have recalled their experiences more accurately than older participants. To help mitigate the likelihood of systematic reporting errors, well-trained interviewers used a standard questionnaire to collect information from all study participants. Additionally, our investigative team worked to make sure that neither the interviewers nor study participants were aware of specific study hypotheses. Furthermore, instruments used in this study to characterize participants’ violence exposure histories [i.e., The Childhood Physical and Sexual Abuse Questionnaire [37], the Demographic Health Survey Questionnaires and Modules: Domestic Violence Module [38] and the WHO Multi-Country Study on Violence Against Women [23] have been shown to be broadly applicable in ascertaining violence exposures in multicultural settings. Additionally, maternal antepartum depression was determined using an instrument that has well-established psychometric properties in diverse Spanish-speaking study populations [42,45,49,50]. Second, we did not have information on the temporal relationship between the onset and end of the reported violence, which precludes the determination of causality in this relationship. In addition, we did not have data on onset of depression or lifetime history of depression. Prospective studies that include clinical evaluation of participants’ mental health status are needed to confirm causal inferences. Third, although 81% of women reporting IPV in the index pregnancy are married and/or lived with their partner, we cannot with certainty claim the perpetrator of IPV was the father of the child. Finally, despite controlling for potential confounders, residual confounding by factors not measured in our study (e.g., witnessing of parental violence, family conflict and social networks) may have influenced reported estimation of associations. Lastly, results from our hospital-based study may not be applicable to the general population of women because women seeking care at INMP are primarily from a low socioeconomic background and may have high-risk pregnancies. However, our study provides data on an important population in Peru, a population of pregnant women that has been documented to have a high burden of social, medical and mental health problems [24,51–54].

The prevalence of childhood physical and sexual abuse and lifetime exposure to physical and sexual abuse by an intimate partner in our study sample is largely consistent with estimates previously reported in the literature [23,24,55–59]. Our finding of increased odds of IPV among women with a history of childhood sexual abuse is consistent with several studies that have documented high risks of violence re-victimization among individuals with a history of childhood sexual abuse [18,60–62]. For example, in their study of pregnant women receiving prenatal care in Soweto, South Africa, Dunkle and colleagues [61] reported that women who were sexually abused as children had an increased risk of being sexually abused by an intimate partner. In their study of urban pregnant women in the US, Nelson et al, reported that women reporting any type of childhood violence were 2.5-fold as likely to be experiencing violence in their study pregnancy (OR = 2.5, 95%CI: 1.8–2.7) [62]. Our findings are also consistent with reports from Arata et al [60] and Trickett et al [18] who reported elevated risks of re-victimization among women who were sexually abused in childhood. Our study extends the existing literature by documenting particularly elevated odds of lifetime risk of physical and sexual abuse by an intimate partner among low-income pregnant women with a history of childhood physical and sexual abuse.

In the present study we found that childhood abuse was associated with 30% higher odds of self-reported poor health status. These findings are consistent with earlier reports. For example, other investigators have reported that survivors of childhood sexual abuse tend to have more negative perceptions of their general health [63,64] and mental health status [65]. In a study of 179 Brazilian women, investigators reported that women who were sexually abused children had poorer perception of health status, including mental health status as compared with non-abused women [65].

In addition to self-reported health, childhood physical and sexual abuse has been shown to be associated with a number of adverse health outcomes in adulthood. For example, childhood sexual abuse has been associated with increased risks of depression [66–68] and suicidal behavior [69–71]. Of note among pregnant women, a history of childhood sexual abuse has been associated with antepartum depression [72,73]. Our finding showing increased odds of antepartum depression among women with a history of childhood physical or sexual abuse (OR = 2.1; 95%CI: 1.6–2.7) is generally consistent with the existing literature. For example, in their study of Israeli pregnant women, Yampolsky et al [74], women with a history of childhood sexual abuse had a 1.5-fold increased risk of depression as compared with women who reported no abuse as a child. In a sample of 357 pregnant US women, Benedict et al. found that history of childhood sexual abuse was associated with more than two-fold increased odds of antepartum depression (OR = 2.4; 95%CI: 1.1–5.3) [75]. Bonomi et al, in a Seattle-based health maintenance organization, found that women with history of psychical and sexual childhood abuse had significantly lower functional health and well-being scores (as measured using the 36-Item Short Form Survey Instrument) (2.32–4.52 points lower), increased odds of fair/poor health (prevalence odds ratio = 1.84; 95%CI: 1.3−2.6) and increased odds of depressive symptoms (prevalence odds ratio = 2.2; 95% CI: 1.8–2.6) (assessed using Center for Epidemiological Studies-Depression Scale) [76]. In sum, our findings and those of others [72,73,76] indicate that childhood abuse has long lasting implications for general and mental health.

Child abuse, a severe early life stressor, is thought to disrupt neurodevelopmental processes that contribute to physical, behavioral and mental health problems later in life. The influences of early life abuse are thought to be modulated, in part, via three neurobiological stress response systems: (1) the serotonin system; (2) the sympathetic nervous system; and (3) the hypothalamic-pituitary-adrenal axis [77–79]. Disruptions to any or all of these systems are known to promote a cascade of physiological, neurochemical, and hormonal changes, which can lead to alterations in brain structure and function and contribute to a myriad of enduring behavioral and cognitive problems[77,78]. For instance, childhood abuse has been linked to behavioral outcomes including internalizing behavioral problems such as limited stress tolerance, anxiety, affective instability, dissociative disturbances, depression and suicidality; as well as externalizing behavioral symptoms including poor impulse control, episodic aggression, substance abuse, attention deficit hyperactivity disorder and conduct disorder [78,80–84]. Childhood abuse has also been associated with a number of cognitive problems including low academic performance and IQ, as well as language, memory, and attention deficits [85]. Taken together, evidence of neuropsychological impairments associated with childhood abuse are consistent with the thesis that early childhood stressors and trauma predispose individuals to subconscious beliefs of unworthiness which may lead to the avoidance of those who truly care and instead a tendency to gravitate towards chaotic relationships [86,87]. In addition findings from structural neuroimaging studies provide evidence of deficits in brain volume, gray and white matter of several regions, most prominently the dorsolateral and ventromedial prefrontal cortex but also hippocampus, amygdala, and corpus callosum among victims of childhood abuse [78]. These data also add biological plausibility to our findings and those of others [2,16,19].

Our study reinforces numerous previous studies confirming high prevalence of childhood abuse [57,88] and high prevalence of lifetime IPV [23,24]. The high frequencies of exposure coupled with the complexity and interrelationships among the types of exposure support arguments for much more systematic, frequent, and intensive efforts to monitor the epidemiology of violence across the life course. Considering that victimization experiences accumulate across the life course, intervention should also target child victims and aim to prevent their future re-victimization. Policies and programs for preventing childhood abuse and its underlying causes are needed. There is a limited but promising body of evidence for preventative intervention programs for childhood abuse such as training in parenting and home visitations [22,89–91]. Careful cross-cultural adaptation of these programs and rigorous evaluation to monitor their impact is warranted. Our findings also suggest that childhood abuse and abuse by an intimate partner in adulthood are factors associated with maternal self-reported health status and antepartum depression. Asking pregnant women during early prenatal care visits about their experience with current and childhood violence may open a discussion about the potential risk of coping with these traumatic events through substance use during the pregnancy. Providing treatment for depression early in pregnancy may significantly improve pregnancy and early child developmental outcomes. The high prevalence of childhood abuse and the enduring effects of early trauma on women’s health warrant concerted global health efforts in preventing violence. Women abused as children are set on a trajectory for subsequent abuse and are a particularly vulnerable population. Public health efforts should be made to prevent childhood abuse, identify women with a history of childhood abuse and provide these women assistance with management of risky health behaviors, mental health issues and ongoing IPV.

## Supporting Information

S1 Table Supplemental Table for Fig. 2. Risk of intimate partner violence (by type of abuse) according to type of childhood abuse*.
(DOCX)
