# Correction: Association of Childhood Physical and Sexual Abuse with Intimate Partner Violence, Poor General Health and Depressive Symptoms among Pregnant Women

**DOI:** 10.1371/journal.pone.0122573

**Published:** 2015-03-23

**Authors:** The PLOS ONE Staff

Fig. 1 is incorrect. Please view the correct Fig. 1 here.

**Fig 1:**
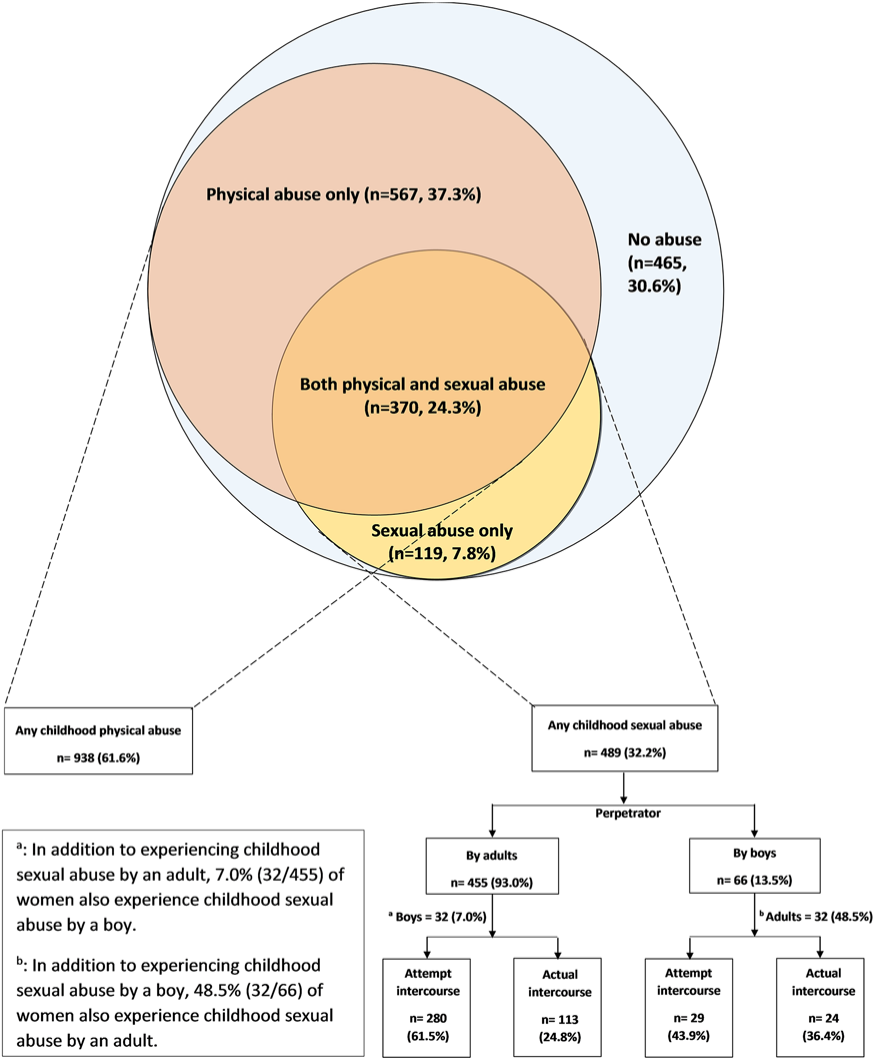
Prevalence of Childhood Physical and Sexual Abuse by Type and Perpetrators among Pregnant Women in Lima, Peru (N = 1,521).
