# Supplementary material for: Association of Childhood Physical and Sexual Abuse with Intimate Partner Violence, Poor General Health and Depressive Symptoms among Pregnant Women
Source: PLoS One. 2015 Jan 30;10(1):e0116609. doi: 10.1371/journal.pone.0116609 (PMC4312043; doi:10.1371/journal.pone.0116609)
Supplement: S1 Table — Risk of intimate partner violence (by type of abuse) according to type of childhood abuse*. (DOCX) [file pone.0116609.s001.docx]

| **Childhood Abuse** | **Lifetime IPV** | |
| --- | --- | --- |
|  | **Type of IPV** | **aOR (95% CI)** |
| **No childhood abuse** | No abuse | 1.00 (Reference) |
| **Childhood physical abuse** | Physical abuse only | **1.62 (1.18, 2.22)** |
|  | Sexual abuse only | 1.23 (0.64, 2.37) |
|  | Physical and sexual abuse | 1.64 (0.94, 2.88) |
| **Childhood sexual abuse** | Physical abuse only | **1.83 (1.10, 3.04)** |
|  | Sexual abuse only | 2.07 (0.82, 5.23) |
|  | Physical and sexual abuse | **3.44 (1.64, 7.22)** |
| **Childhood physical and sexual abuse** | Physical abuse only | **2.69 (1.89, 3.84)** |
|  | Sexual abuse only | **4.82 (2.62, 8.87)** |
|  | Physical and sexual abuse | **6.88 (4.03, 11.76)** |

CI = confidence interval; aOR = adjusted odds ratio; Bold = statistically significant values.

*Odd ratios and 95% confidence intervals are from a multinomial logistic regression model that included adjustment for maternal age (years), education (years), employment status (yes vs. no), parity (nulliparous vs. multiparous) and difficulty paying for the very basics (very hard or hard, somewhat hard, not very hard)
